# Homœopathy

**Published:** 1837-01-01

**Authors:** 


### HOMŒOPATHY.

I. A Practical View of Homœopathy; being an Address to British Practitioners on the general Applicability and superior Efficacy of the Homœopathic Method in the Treatment of Disease. With Cases. By *Stephen Simpson*, M.D. late Resident Practitioner at Rome. Octavo, pp. 350. London, 1836.

II. Animal Magnetism and Homœopathy, &c. By *Edwin Lee*, M.R.C.S. formerly House-Surgeon to St. George's Hospital. Octavo, stitched, pp. 40. London, 1835.

III. Exposition of Homœopathy. By Dr. *Bureaud Riofrey*.

IV. Articles on Homœopathy in the Medical Gazette. Nos. 467 and 469.

Every age has been distinguished for its gross credulity in matters relating to the practice of medicine. In the infancy of the world, or in the absence of civilization, the ignorance that must exist with respect to the constitution of the animal frame, explains and extenuates the belief in absurdities however great, the credit given to impostures however gross, relating to the operation of material or of immaterial agencies upon it. Amulets and charms, drugs selected from fanciful analogies of shape, or colour, or smell, the most inert things when consecrated by dogmatism or by superstition, have been found in the materia medica of every rude and every half-informed nation, and have only been banished from it by the gradual march of knowledge and the slow process of enlightenment.

The student of history cannot be surprised at the display of popular prejudice or popular fanaticism. We have felt no astonishment at the exhibitions of our own day—and the rise and fall of successive delusions within our observation have only served to shew that man is the same animal that he ever was, and that, despite of the intellectual character of the nineteenth century, it still contains rogues enough to humbug the public, and fools enough to make it worth the while of rogues to humbug them.

Yet even cynicism must confess, that, in prevalent delusions, all the deluded are not of necessity either rogues or fools. There are many, of enthusiastic dispositions, who are led away by novelty and caught by specious arguments, yet are neither absolutely weak in mind nor wrong in purpose. Every-day experience shews us individuals amongst our acquaintance or our friends, who display this intellectual singularity; and the combination of vigour and weakness, of general right reasoning and of a special folly, is at once curious and instructive. Such a combination is incompatible with great soundness of mind or much depth of information; but observation shews that it is consistent with brilliant intellectual qualities, with wit, eloquence, or imagination.

When homœopathy was first promulgated, the universal derision with which it was received in this country, inclined us to hope that the English public would not be much bitten by the rabid advocates of such mystical foolery. That hope has been scarcely disappointed, although it must be owned that a few persons, with some pretensions to science, have turned proselytes or even professors. Their number, however, has been marvellously small, their names and influence far from imposing, and their efforts have only served to crown themselves with ridicule, and, by provoking argument and free discussion, to accelerate the fall of what it would be laughable to call a system.

The basis of the science of medicine is fact; the science itself should be strictly one of induction. That induction may be more rigorous or less, in accordance with the nature of the facts that it is founded on.

An individual presents phenomena, which in their aggregate constitute disease. We are anxious to learn what that disease actually is, what those phenomena really represent. Death enables us to do this, for then, by dissection, we find certain alterations of the textures of an organ, with the natural state of which we had previously, by appropriate investigations, become conversant. One such observation is compared with many others, and the sum of their agreements and their differences constitutes our knowledge of the disease. All this is a strict and logical process of induction, a process which has led us to most of what we positively know in physics and philosophy.

We have learnt, or seemed to learn, the real nature of that disease. Our next object is to cure it. To do this, we must ascertain the properties of any substance which exerts an influence upon the organs of the body. We re-commence a series of inductive observations. We exhibit the drug, if it be a drug, that forms the subject of experiment, and note the effect that it produces. To render the observation precise, the operation of the drug on the healthy organ should be first found, and subsequently its effect on the diseased one should be tested. Again, one observation is corrected by comparison with others, and finally we obtain as the result of our researches the effects of the medicine in health and in disease. Such inquiries repeated by innumerable persons and for many years have led to the construction of our materia medica.

Pathology, then, and the doctrines of our materia medica, our knowledge of the nature of disease and of the means of treating it, are both essentially founded on fact, reared by cumulative observation, and operations of induction. That there are errors is not wonderful, for cumulative observations must be open to them. But those errors are continually diminishing, and fresh experiments and fresh observations which confirm the true, are gradually ridding medicine of much of the false. We say much, for many fallacies there always must be in a science, the subject of whose inquiries is not a physical entity which never changes, but the operations of life, continually agitated by disturbing circumstances so numerous and so subtle as to baffle positive calculations.

To those who comprehend the manner in which medicine has been elevated to a science, and who know that all its most valuable portions are essentially facts, the proposal of a new scheme, which sets aside all former observations, contradicts all previous experience, and upsets, not only theory, which may be wrong, but facts too, which cannot—a scheme, in short, which insulting rational belief as folly, demands the most implicit credulity from its votaries—to all of a philosophical character, such a scheme must appear the height of human folly.

Hahneman, its inventor, knew full well that inductive science and homœopathy were incompatible, and he declared war against induction. He did not hope, he could not hope, to overthrow it, but he probably knew that the fools, like the devils, are named legion, and he appealed to them. This may seem a caricature, but it is not—it is the very essence of homœopathy. Dr. Bureaud Riofrey, a very intelligent French physician, residing in London, has given, in a paper read before the Westminster Society, some extracts from Hahneman's Organon, which establish this point completely. And indeed a little consideration would shew that Hahneman could not do otherwise than reject facts which would oppose his dogmas.

"Homœopathy acknowledges no organic alterations. Hahneman says, that in order to gratify our folly, diseases cannot cease to be dynamic aberrations that *our spiritual life feels*; that is to say *immaterial changes* in ourselves. (Organon, p. 15.)"

As Hahneman denies that diseases are material, he denies too that its causes are so; nay he says they cannot be so. As neither diseases nor their causes are material, men may reasonably inquire what they are. The reply is clear. They are partial alterations of *dynamic* life. Can any thing be more lucid.

"Diseases are not, and cannot be mechanical, or chemical change of the material substance of the body: they do not depend on a morbid material principle; they are merely partial alterations of *dynamic* life." (Organon, 129.)

Dr. Bureaud remarks in reference to this brilliant and perspicuous hypothesis:—

"Therefore, gentlemen, we return towards ontology; diseases are separated from the organs, effects are without cause, and our senses are deceived by the phenomena that take place. It is no longer necessary to study and to learn the influence of modifications, of the agents of nature on each of our organs; life and disease are phenomena independent of the body, and all manifestations are produced in such a manner as the profane cannot yet appreciate. Homœopathists only acknowledge dynamic affections: and although this dynamia is but the representation of material effects, homœpathists still maintain that matter has no share in the manifestations." 2.

Dr. Johnson said that he liked a good hater. We must say that we like a good theorist. Of what use is it to propose a snivelling scheme, which any body is capable of understanding? The very height of intellect is to devise what other intellects are neither able to devise nor comprehend. How beautiful is the contrast between the doctrine of disease being a partial alteration of dynamic life, and that poor grovelling induction, drawn from observation, and from the use of men's senses, which seeing certain organic changes correspond uniformly with certain symptoms, terms those symptoms the disease and those changes its material causes. Simplicity has been thought to be the merit of the science of causation;—how falsely this new philosophy proves.

Hahneman proceeds to declare that symptoms are the external image of the internal disease, and that they alone merit the patient's attention. A slight metaphysical difficulty occurs, which it would be well, perhaps, to get rid of. We know there are symptoms, from observation. A patient, for instance, says he has a sharp pain in his side. That pain is not an object of sense to us. He breathes quickly and imperfectly, for his face is blue. These are objects of sense. He suddenly dies. The lung, which we know to be the organ of respiration, is found choked with serum, lymph, and pus. This is an object of sense too, and the ignorant physician, taking it with the symptoms, concludes this was the cause of the pain and the dyspnœa. But the homœopathist says no. That is material, but diseases are immaterial, therefore that is not the cause of the symptoms. The difficulty, then, is to conceive how material symptoms, hurried respiration, and asphyxia, can have an immaterial cause—and how material alterations of the respiratory organs shall not be the cause of symptoms. A reasoner not imbued with the true spirit of homœopathy finds it hard to believe, that a certain mode of respiration shall be the consequence of a lung healthy, and that an altered mode of respiration shall not follow from a lung altered. But this captious disposition to inquiry is not favourable to the reception of homœopathic truths.

If disease be only symptoms, there are some things that require explanation. Dr. Bureaud ignorantly asks:—

"But let us now inquire how homœopathists act when the symptoms of an existing evil are not evident. When an individual is bitten by a mad dog, will they prevent the wound being cauterised, when there are no other means of stopping the progress of the virus? Is there no disease because there is no symptom? and will they wait till the patient is attacked with the dreadful convulsions attendant on this fearful disease, to administer a quadrillioneth part of a grain of belladonna, of hyoscyamus, or stramonium? Can it then be said that the *tolle causam* of ancient medicine is a chimera? that the disease in this case, as in a thousand others, is but a dynamic aberration of *our spiritual life?* Will homœopathists then assert that they have not seen the cause, as they say they have not seen Vanhelmont's thorn. Will they then inquire what quantity of rabid virus has been absorbed, in the same manner as they ask what quantity of syphilitic virus has penetrated?" 3.

Dr. Bureaud is an unreasonable man. What is the utility of a mystical truth, unless it is to put down sophistical scepticism, unless it is to silence specious objections? It seems more consonant with fact to believe that a boy being bitten by a rabid dog, and afterwards becoming rabid, has received a virus, which has remained for a period quiescent—but can reason be opposed to authority—are facts to be weighed against the assertions of the great Hahneman?

This prophet, like his illustrious and ingenious predecessor Mahomet, spent many years in reflection. We have seen that symptoms have no causes, save a dynamic alteration of our spiritual life. One great advantage of homœopathy is, that it not only contradicts all facts, but it is enabled to contradict itself, without being in any degree the worse. Thus, after twelve years of intense meditation, Hahneman determined that diseases are acute or chronic, and that chronic diseases *have* a cause—syphilis—sycosis—*or* the itch.

"This—the itch," says the philosopher, "is the only real, fundamental, and productive cause of all the morbid forms known by the names of weakness, nervousness, hysteria, hypochondria, mania, melancholy, epilepsy, spasms of all kinds, rickets, caries, cancer, fungus hæmatodes, gout, hemorrhoids, jaundice, cyanosis, dropsy, amenorrhea, gastrorrhagy, asthenia, sterility, cataract, gravel, paralysis, and pains of all kinds, &c. &c." (Org. p. 183.) 3.

A great mind strikes out a path for itself. Who would have imagined that the itch was the cause of diseases apparently so dissimilar as cancer, cataract, and the gravel—of asthenia and gout—rickets and pains of *all* kinds? Here is indeed the evidence of genius. Yet sublime conceptions have sometimes flowed from the most trivial causes. Perhaps this magnificent idea may be traced to the sage having suffered from the "Scotch fiddle" himself. We diffidently offer it as the only explanation that occurs to us, of this immortal discovery. We pause. It is useless to pursue a doctrine which is made up of such marvellous principles that it is not necessary for it to be in any way consistent with itself, with fact, or with anything. It is better at once to receive the whole science at a swallow, and extend to it that implicit belief which it requires. It is of no avail trying to make it consistent with reason, and reason therefore should succumb.

Fortunately, Hahnemann was not a mere speculator. His object was not simply to divulge a scientific revelation. He was anxious to benefit humanity substantially as well as to delight it, and he has given us such a system of pharmacy and therapeutics, as is worthy of the pathology on which it is engrafted.

"Having in 1790 taken some bark, which produced, as he states, paroxysms of intermittent fever, he was struck with the circumstance that the substance employed for the cure of intermittents should occasion a similar disease in a healthy person. This led to the inference that substances which produce certain symptoms in healthy individuals can remove these symptoms when induced by other causes; hence a fundamental point of the doctrine, that diseases are cured only by medicines which have the power of causing similar diseases in healthy persons: *Similia similibus curantur.*" 23.

It might appear odd that cures have taken place before this truth was discovered. The explanation is obvious and conclusive.

"To prove that many of the cures hitherto effected have been so by the chance employment of homœopathic means, several instances are brought forward, among which are, that rose-water cures ophthalmy only because it has the power of causing a kind of ophthalmy. In like manner bark cures intermittents, because it occasions these diseases: ipecacuanha arrests fluxions of blood, only because it possesses the faculty of exciting hæmorrhage; generous wines, in small doses, cure homœopathically inflammatory fever; hyoscyamus could not cure spasms resembling epilepsy, if it had not the power of exciting convulsions; the same remedy could not have cured a case of mania from jealousy, if it did not occasion mania and jealousy in healthy individuals." 25.

The facts are as ingenious as the explanations are convincing. How many times must hyoscyamus have caused jealousy, and yet we have never noticed it. Ipecacuanha too must, on countless occasions, have given rise to alarming hæmorrhages, without attracting the observation either of the patient or physician. Ophthalmia from rose-water must be of daily occurrence, and it must possess the singular property of blinding not only those who have it, but those also who try to see it.

Our therapeutics are indeed simple. Pneumonia is a disease of inflammation. It must be treated by producing more inflammation. Induced weakness cannot rationally be supposed to master a disease of strength. Devils are driven out by Beelzebub, because he is the prince of them.

"It is the extreme of ignorance to think of curing pneumonia by bleeding; for the rapid operation of vital strength would in most cases cease unaided; but it might also overpower the *vis medicatrix naturæ*, for it is a general rule that strength overpowers weakness. Instead, therefore, of arresting the operation of the vital force, which Hahnemann terms blind and vulgar nature, his great discovery is to *increase* it by artificial means, by an analogous and artificial disease, as nearly as possible resembling the original one." 4.

If the principles of Hahnemann's therapeutics equal those of his pathology, the details of his materia medica are not unworthy of both. The principle on which a drug is given, is the immortal one—similia similibus curantur. The drug produces a disease which puts the existing one to flight. The first object is, therefore, to ascertain, by experiments on healthy people the effects of various medicines, in other words, the disorders which they cause. This has been done with the success that migbt be expected to crown the efforts of one, whose genius has discovered the memorable fact, that the itch is the cause of most chronic maladies.

It is difficult to determine how Hahnemann discovered the mighty truth, that the potency of medicines increases in the ratio of the decrease of the dose. It is humiliating to reflect that the world should so long have believed in the opposite pernicious error; and it is impossible to account, for what seems the fact, that poisons destroy those people who take them not in little but in large quantities. No reasonable man would set the evidence of his senses against the assertions of Hahnemann, and we receive with the submission they deserve the following revelations.

"The effects of medicinal substances," says the philosopher, "are two-fold, viz. primitive, as the violent action produced by large quantities of certain drugs; purgation, sweating, &c.; and secondary, or homœopathic, in which the action is determined towards the diseased part; the active properties becoming more developed in proportion to the minuteness of the dose: in fact, homœopathists are cautioned against too minute a subdivision of the medicine, lest it should become so energetic as to give rise to dangerous symptoms." 27.

It is not the mere subdivision of the medicine that so alarmingly augments its strength—it is also the shake that the adept gives it. We tremble when we think what mischief has been done by the incautious direction too frequently labelled on a draught, " when taken to be well shaken." Hear Hahnemann himself.

" Besides, the homœopathic medicament acquires at each division or dilution an extraordinary degree of power by the friction or the shock imparted to it, as means of developing the inherent virtues of medicines, unknown before me, and which is so energetic, that of late, experience has obliged me to shake only twice, whereas formerly I prescribed ten shakes to each dilution." 27.

What a train of reasoning and experiments, what profound sagacity must have been expended by the *Maestro*, in determining the number of the shakes. It may indeed be said of him, now that he has limited them to only two, that he is " no great shakes" himself. Forbid it Heaven that he should be so, for then he would literally " be the death of us."

Whichever way we turn, we find some popular error corrected, some established fact disproved. We have learnt that the danger of doses of medicine depends on their infinite minuteness. We shall find that substances always deemed inert are possessed, in homœopathic doses, of potent and peculiar properties. In a former article in this Journal we made some extracts from Dr. Quin's edition of Hahnemann's Pharmacopœia, and astonished our readers, with the description of the properties of the common " fever-few." We need not now repeat them, for probably the profession is well acquainted with them. Yet we may remark, that some of them are worthy of the science. Dentium elongatio, lengthening of the teeth, was found to result from an infinitesimal dose of the drug, and other effects equally peculiar, exhibited its powers.

" Gold, silver, platina, charcoal, are without action on man in their ordinary state, but from the continued trituration of a grain of gold with an hundred grains of powdered sugar, there results a preparation which has already great medicinal virtue. If a grain of this mixture be taken and triturated with another hundred grains of sugar, and if this process be continued until each grain of the ultimate preparation contains a quadrillionth part of the grain of gold, we shall then have a medicament in which the medicinal virtue of the gold is so much developed, that it will be sufficient to take a grain, place it in a phial, and cause the air from it to be breathed for a few instants by a melancholy individual, in whom the disgust of life is carried so far as to incline to suicide, in order that, an hour afterwards, this person be delivered from his evil demon, and restored to his taste for life." 28.

If smelling a quadrillionth of a grain of gold can effect so much, what would a quadrillionth of *it* do? We cannot contemplate with calmness the consequences of the square root of a quadrillionth of a grain. It would poison a regiment. Our contemporary, the Medical Gazette, has been perusing with much diligence the materia medica of Hahnemann, and has made some extracts from it, on the properties of charcoal and of gold, which tend to increase our admiration of the homœopaths. Their scientific investigations are of a very original and pleasing character, and strongly remind us of the researches of Philosopher Square and Molly on " the eternal fitness of things." Imagine a homœopath (Gersdorff is his name) eating one night a prescribed supper, consisting of French beans, green peas, and carrots, and swallowing the millionth of a grain of charcoal. He retires to rest, and abstracting his attention from all else, he accurately notes the effects of that not trivial dose. Soon he hears with delight:—

308. Emission sourde de vents presque inodores, humides, et chaudes.

In an hour and a half, he not only hears but he smells the gale.

312. Vents ayant un odeur putride.

The odeur putride is not offensive to the homœopathic nose, for it tells of the triumph of the art. The charcoal, however, has not yet done all, or nearly all, it is capable of doing. It works whilst the homœopath slumbers, and there results ;—

375. Erection continuelle pendant la nuit, sans sensations ou idées voluptueuses.

When the homœopath wakes, it is only to philosophize, for instantly there is—

312. Enorme emission de vents sans odeur.

How charming ! But he has not done. A female homœopath, the representative of Molly, has had her commissions to execute, and before the two compare their notes, Gersdorff, remembering the erection continuelle pendant la nuit, hints the fact to his fair associate, but—

376. Il ne peut meme ètre excité par des idées lascives.

Let us leave Gersdorff, in his vain attempts to obtain a repetition of the "érection continuelle," and turn to the experimentress. She has taken a millionth of a grain of charcoal too, and probably eaten of the same windy diet of beans and pease. She does not however generate " vents." Gersdorff was the Œolus, and furnished them. Had both been equally *ventoses*, the late storm would have been nothing to the turmoil " 'twixt the sheets." The lady modestly complains of an—

378. Ardeur dans les parties genitales.

This increases, and soon there is—

377. Forte excoriation aux parties genitales.

We drop the curtain on the two philosophers. We are told, indeed, of various "pollutions," "pertes de semence," and "emissions de vents," but as the Allopaths have not arrived at the happy pitch of disregarding common decency and common sense, we must, in deference to such prejudices, quit the subject.

Homœopathy, such as we have depicted it, founded on such reasoning, supported by such facts, has been put, in some quarters, to the test of practical experience. The results might be anticipated.

" A German homœopathist, practising in Russia, was invested by the Grand Duke Michael with full powers to prove, if possible, by a comparison of facts, the advantages of homœopathic measures over the ordinary modes of treatment; and a certain number of patients in the wards of a military hospital were entrusted to his care. At the expiration of two months, however, he was not permitted to proceed further; for, in comparing results, it was seen that within this period, of four hundred and fifty-seven patients treated by the ordinary means, three hundred and sixty-four, or three-fourths, were cured, and none died ; whereas, by the homœopathic method, tried on one hundred and twenty-eight patients, one half only were cured, and five had died." 30.

In consequence of this and other trials, the Russian government looked on homœopathy as humbug, and published the results.

Such is a slight sketch of the principles and the practice of homœopathy. It may be shortly characterised as the most impudent insult on the common sense of men, that has ever been offered to it in any age or in any country.*

* Dr. Simpson's work, professing to exhibit new views of homœopathy, will be found noticed separately, in the department of the present Number appropriated to "Miscellaneous" matters.

